# A Candidate Gene for a Biological Marker of Schizophrenia in Mice

**DOI:** 10.1371/journal.pbio.0050320

**Published:** 2007-11-13

**Authors:** Liza Gross

Little more than 35 years ago, the notion that domineering mothers could induce schizophrenia in their children with their overbearing, identity-extinguishing behavior still held sway among psychiatrists. By the mid-1970s, the “schizophrenogenic”-mother hypothesis lost favor to genetic explanations of the disease. Researchers now believe that multiple genes interact with environmental factors, such as birth trauma or perinatal infection, to cause schizophrenia. And though defects in several chromosomes have been linked to schizophrenia, the hunt for candidate genes continues.

Although the disease's trademark delusions, hallucinations, social withdrawal, and emotional “flattening” typically emerge in late adolescence or early adulthood, schizophrenia appears to be a developmental rather than a degenerative disorder. Evidence from human patients suggests that disrupted fatty acid metabolism may promote susceptibility, and efforts to understand how different drugs control schizophrenia's symptoms have implicated several brain neurotransmitters, including dopamine, glutamate, and serotonin. Increasing evidence also suggests that defective glial cells—which not only support other brain cells but also control their activity—may cause the disease.

In a new study, Akiko Watanabe et al. identify a candidate gene that links abnormal lipid metabolism to glial and developmental theories of schizophrenia. The gene, called *Fabp7* (*Fatty acid binding protein 7*), encodes a protein that helps the essential fatty acid docosahexaenoic acid (DHA) assume its proper shape. It also has functional links to the glutamate receptor N-methyl-D-aspartic acid (NMDA).

In schizophrenia, deficits in the brain's sensory “gating” mechanisms, which prevent sensory overload, disrupt a person's ability to perceive or process information, leading in turn to abnormal thoughts (hearing voices) or actions (frenetic pacing or repetitive hand gestures). One such gating mechanism, called prepulse inhibition (PPI), can be studied by measuring the acoustic startle reflex in mice. In normal mice, PPI suppresses the startle reflex in response to an unexpected sound that is immediately preceded by a lower-intensity sound. PPI, which is common in schizophrenia, serves as a measurable, internal biological marker for the disease. Researchers hope that identifying the genes responsible for PPI will point to genes that increase risk for schizophrenia.

Toward that end, Watanabe et al. screened four mouse strains for differences in their PPI responses. The researchers selected offspring of the two strains with the highest (the B6 strain) and lowest (C3) responses for experimental testing and genetic analysis. Working with over 1,000 mice, they measured the amplitude and latency (delay between stimulus and maximum response) of the animals' acoustic startle reflex and their PPI over a range of prepulse sound levels.

To identify the gene variants (or alleles) that contribute to a complex, or quantitative, trait like schizophrenia (or its proxy, PPI), the researchers used quantitative trait loci (QTL) analysis, a statistical method that estimates the likelihood that specific loci (called quantitative loci) affect the trait (or phenotype) under study. The researchers collected genetic data from all 1,010 second-generation mice using 80 markers called “microsatellites,” short stretches of repeat DNA sequences with high mutation rates that provide a handy way of assigning individuals to different groups based on their microsatellite genotypes. They sorted mice into 21 groups with about 50 individuals each, and then measured their phenotypes (PPI at the different sound levels, acoustic startle reflex, and latency).

**Figure:**
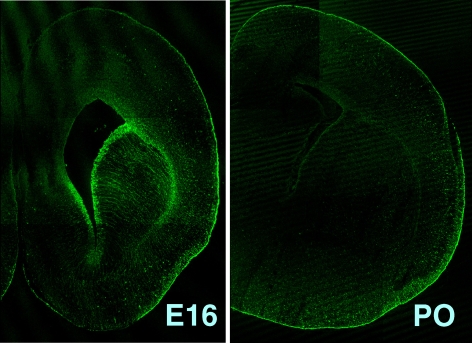
Expression of Fabp7 protein in mouse brains at embryonic day 16 (left) and postnatal day 0 (right). At both stages, Fabp7 is strongly expressed in the ventricular zone and radial glia, where neurogenesis is prominent.

Using a computational model designed to detect QTL signals across the whole genome, the researchers identified six chromosomes with potential contributions to PPI, including a strong candidate on Chromosome 10. Next, they used a different computational model to extract more detailed information on the six chromosomes. They detected a very strong signal for PPI on the Chromosome 10 region that harbors the gene encoding the brain-type fatty acid binding protein (Fabp7).

Fabp7 shows strong binding affinity for the fatty acid DHA (disrupted DHA metabolism has been implicated in schizophrenia), and is required to maintain stem and progenitor cell populations in the developing rat cortex. It also participates in NMDA-mediated neuron signaling—which influences both PPI response and schizophrenia. Watanabe et al. used several experimental approaches to explore the gene's functional link to PPI.

First, they examined the gene's transcript levels in the C3 and B6 mouse strains across different brain regions at discrete developmental time points: C3 animals (which had the lowest PPI response) had higher *Fabp7* expression levels, with just a few exceptions (including the premature frontal cortex). By generating “knockout” mice with no functional Fabp7 proteins, they demonstrated a link between *Fabp7* and reduced PPI and startle response latency. Young knockout mice also showed a marked reduction both in the number of embryonic stem and progenitor cells and in the number of proliferating cells, suggesting that *Fabp7* disrupts new brain cell production. By performing what's known as a “complementation test,” the researchers provided evidence for *Fabp7* as one of the genes responsible for the PPI–QTL signal on Chromosome 10. And when they analyzed tissue from the brains of deceased patients with schizophrenia and normal individuals, they found increased *Fabp7* transcript levels in the affected tissue.

Taken together, these results indicate that *Fabp7* is responsible for differences in the animals' PPI responses and that the gene plays a vital role in brain development. Given the link between in utero malnutrition and increased risk for schizophrenia, the researchers reason that dysregulation of *Fabp7* and lipid metabolism during development may cause long-term changes in gene expression, explaining an excess of *Fabp7* in the schizophrenic cortex. Whether *Fabp7* operates alone or with other genes on Chromosome 10 must be explored in future studies. And while no methods to prevent schizophrenia exist, the researchers recommend designing cohort studies to find out whether supplementing the diets of pregnant mothers in high-risk families with DHA proves beneficial.

